# Phyllospheric fungal diversity in decomposing larch leaf litter: a comparative study of epiphytic and endophytic fungi

**DOI:** 10.3389/fmicb.2024.1489889

**Published:** 2024-11-22

**Authors:** Hong Pan, Dan Wei, Libin Yang, Xiaoyu Fu, Daoguang Zhu, Xinming Lu, Siyuan Liu, Yongzhi Liu

**Affiliations:** ^1^Key Laboratory of Biodiversity, Institute of Natural Resources and Ecology, Heilongjiang Academy of Sciences, Harbin, China; ^2^Heilongjiang Huzhong National Nature Reserve, Huzhong, Greater Khingan Mountains Region, China

**Keywords:** phyllospheric fungi, leaf litter decomposition, community diversity, nutrient loss, *Larix gmelinii*, cold temperate forest

## Abstract

**Introduction:**

Epiphytic and endophytic fungi are primary decomposers of forest litter due to their complex species composition and metabolic functions. To clarify the community diversity of phyllospheric fungi and to explore nutrient loss and the role of fungal decomposition, we conducted a study on the decomposition of leaf litter during the 1-year decomposition of *Larix gmelinii* in the cold temperate zone.

**Methods:**

Fungal diversity data were characterized via Single Molecule Sequencing (based on the Sequel II Sequencing System) and statistical analyses in R.

**Results and discussion:**

Our findings revealed the presence of 11 known fungal phyla and 29 dominant genera in the larch litter of Greater Khingan. Among these, Basidiomycota and *Leucosporidium* were dominant in the epiphytic environment, while Ascomycota and *Exutisphaerella* dominated the endophytic environment. In the early periods of decomposition, phyllospheric fungi became the primary colonizers during litter decomposition by adjusting their life strategies to transition to saprophytic or pathogenic metabolic processes. During decomposition, significant differences in alpha diversity were observed between endophytes and epiphytes. Correlation analysis between these fungi and biological factors revealed a strong relationship between cellulose loss in leaves and the return of N, P, and K. This indicated that the combined biological effects of nutrients, aminosugars, and plant fibers strongly explained changes in community structure. Our results also revealed a significant clustering effect between fungi and biological factors, reflecting the important role of phyllospheric functional fungal communities in carbon fluctuations, cellulose decomposition, and the enrichment of P and K in leaf litter. In summary, this study offers insights into ecosystem processes and nutrient cycling within cold temperate forests, with potential applications for understanding global carbon dynamics.

## Introduction

Litter is defined as the sum of all debris returned to the ground by fallen vegetation, including leaves, branches, fruits, withered herbs, roots, and other materials, with leaf litter comprising more than 50% of the total (Guo et al., 2015). Leaf litter is a major source of nutrients for plants, microorganisms, and soil animals. Larch litter is primarily comprised of leaf litter, which is mainly decomposed by microorganisms. During decomposition, the released nutrients can be transferred between different types of litter, eventually entering the soil through bio-organic metabolism, where they are reabsorbed and utilized by plants (Leppert et al., 2017).

Currently, leaf litter decomposition is a major focus in forest ecology research, with studies primarily examining the effects of climate and site conditions on decomposition (Xing et al., 2024; Sagi and Hawlena, 2023), the response of decomposition processes to nitrogen deposition (Wrightson et al., 2024; Song et al., 2024), leaf litter mass loss and nutrient release (primarily carbon output) (Zhang et al., 2024; Piazza et al., 2024), decomposition rates and limiting factors (Nakanishi and Tsuyuzaki, 2024; Lin et al., 2024), and the roles of soil animals and soil microbial functions (Contos et al., 2024; Wang et al., 2024). Nevertheless, little is known regarding how fungal communities affect and regulate leaf litter decomposition. Phyllospheric fungi are widely recognized as crucial decomposers, playing a key role in nutrient cycling by breaking down organic matter in forest ecosystems (Yan et al., 2010; Sun et al., 2021). After plant leaf fall, fungal hyphae invade the tissue and secrete various extracellular enzymes that convert hard-to-decompose sugars and phenolic substances into small, soluble organic molecules, making the leaf litter easier to decompose further (Waring, 2013). Additionally, the succession of fungal communities also influences the decomposition process of litter. In the early periods of litter decomposition, leaf mass loss is primarily driven by Basidiomycetes and Ascomycetes, which effectively decompose carbohydrates (Sun et al., 2021). As decomposition progresses, the dominant fungal groups shift to saprophytic fungi that can utilize more resistant substances (Chakraborty et al., 2020). This succession process highlights the functional differences in fungal community structure at various periods of litter decomposition, a phenomenon mostly observed in tropical, subtropical, and mid-temperate regions, with limited research in cold temperate climates (Marian et al., 2019; Lin et al., 2019; Bahnmann et al., 2018). Although phyllospheric fungi play a crucial role in initiating leaf litter decomposition and nutrient release in global forest ecosystems (Carnicer et al., 2015), microbial distribution is more strongly influenced by vegetation types and climatic conditions at the micro-scale (Fukasawa et al., 2021; Tian et al., 2018; Wu et al., 2021). Therefore, the species and functional differences exhibited by fungal communities during leaf litter decomposition in cold temperate climates are particularly worthy of attention.

Larch is the dominant vegetation type in the cold temperate forest of the Greater Khingan Range. Compared to tropical, subtropical, and temperate zones, this cold temperate larch forest is an especially sensitive northern ecosystem affected by climate change. In response to global climate shifts, the larch ecosystem adjusts its water, heat, and carbon exchange with the atmosphere to mitigate warming, acting as a crucial barrier in supporting and stabilizing the healthy development of northern forest ecosystems. Currently, research on nutrient loss from cold temperate forest litter at mid to high latitudes remains limited, and the complex interactions between fungal communities involved in leaf litter decomposition and nutrient loss in these forests have been largely overlooked. Previous studies have primarily focused on the effects of endophytic fungi or specific functional genera on litter nutrient and quality loss, restricting the scope of fungal research in natural conditions and limiting the depth of these studies. Therefore, building on prior research, we offer the following novel insights: (1) high-throughput sequencing enables more accurate and comprehensive identification of in situ fungi in the field, providing a detailed understanding of phyllospheric fungal community structure and dynamic evolution across different stages of litter decomposition; (2) we link fungal diversity to nutrient flow, comparing the diversity of epiphytic and endophytic fungi and examining their correlations with nutrient loss in needle leaf litter; and (3) we further investigate the functional differences between epiphytic and endophytic fungi, assessing their roles in larch leaf litter decomposition and nutrient cycling. Our findings reveal a more intricate picture of the role of phyllospheric fungi in litter decomposition, providing a theoretical foundation for future research into the impact of fungal communities on material decomposition in cold temperate forests amid climate change.

## Materials and methods

### Experimental area and sample collection

This study was conducted in the Heilongjiang Huzhong National Nature Reserve (51°49′01″-51°49′19″N, 122°59′33″-123°00′03″E) in the Greater Khingan Range Region of China. The terrain in this area is gentle, with a mean elevation ranging from 847 to 974 m. The mean annual temperature is −4°C, with an average annual precipitation of 458.3 mm, mean annual relative humidity of 71%, and mean annual evaporation of 911 mm. This region represents a global cold temperate climate and boreal forest ecosystem, with larch as the primary zonal vegetation, making it a key area sensitive to global ecological change. Here, the research team has established a permanent large-scale plot (25 hm^2^) in the cold temperate larch forest, where extensive studies on forest biodiversity, coexistence mechanisms, climate change, and ecological response have been conducted, yielding significant discoveries and progress over the past decade.

The experimental area was located within a 25-hectare larch forest plot in the Cold Temperate Zone of China, which is part of the Chinese Forest Biodiversity Monitoring Network (CForBio) and the Center for Tropical Forest Science - Forest Global Earth Observatory (CTFS-Forest GEO). A 20 m × 20 m sample plot was established, with three sampling points arranged within the plot. To investigate the initial decomposition process of leaf litter, a one-year decomposition period was established, with samples collected at five stages: (1) Green Leaves Period (GL): In August 2022, healthy green leaves were randomly collected as a control; (2) Withered Leaves Period (WL): In early September 2022, unfallen withered leaves were randomly collected; (3) Fallen Leaves Period Phase I (FLI): In late September 2022, naturally fallen leaves were gathered using a collection box; (4) Fallen Leaves Period Phase II (FLII): FLI leaves were placed in litterbags, fixed on the soil surface at the original larch location in late September 2022, and collected in May 2023; (5) Fallen Leaves Period Phase III (FLIII): In September 2023, leaves in litterbags were collected. Three parallel samples were collected at each sampling point during each period, resulting in a total of 45 leaf samples, which were packaged, labeled, and brought back to the laboratory. After mixing the parallel samples, 15 test samples were obtained. One part of these samples was used for gene sequencing of epiphytic fungi (EpiF) and endophytic fungi (EndF), whereas the other part was used for detecting biological factors in coniferous leaves, with each sample tested in triplicate.

### DNA extraction

The DNA of larch phyllospheric microorganisms was extracted using the TGuide S96 Magnetic Soil/Stool DNA Kit (DP812, Tiangen Biotech, China) according to the manufacturer's instructions. Following vortex mixing of the pre-treated sample, it was heated at 70°C for RZ pyrolysis, yielding 500 μl of supernatant. The lysed samples were then purified using magnetic bead-specific adsorption (twice), followed by RD protein removal and TB buffer elution, preparing the samples for use. The magnetic bead recovery efficiency for nucleic acid samples was over 95%.

### Sequence amplicon and gene library construction

The ITS rRNA full-length gene was amplified from the genomic DNA extracted from each sample using the ITS1F (5′-CTTGGTCATTTAGAGGAAGTAA-3′) and ITS4 (5′-TCCTCCGCTTATTGATATGC-3′) universal primer set, with a Veriti^®^ Thermal Cycler (Veriti 96-well, Applied Biosystems Inc., USA).

The reaction mixture consisted of the following: KOD One^TM^ PCR Master Mix (KMM-101, TOYOBO Life Science, Japan) 15 μL; Forward Primer (5 μmol/L) 0.9 μL; Reverse Primer (5 μmol/L) 0.9 μL; Template DNA (10 ng/μL) 1.5 μL and nuclease-free water 11.7 μL, bringing the total volume to 30 μL.

PCR amplification of the ITS full-length sequence was performed with the following thermal cycling conditions: initial denaturation at 95°C for 5 min, followed by eight cycles of denaturation at 95°C for 30 seconds, annealing at 55°C for 30 seconds, and extension at 72°C for 45 seconds. This was followed by 24 cycles of denaturation at 95°C for 30 seconds, annealing at 60°C for 30 seconds, and extension at 72°C for 45 seconds, with a final extension at 72°C for 10 min. The reaction was then stopped at 4°C.

After PCR amplification, the amplicons were first detected using 1.8% agarose gel electrophoresis. If the main electrophoretic band was clear with minimal impurities, samples were mixed in a 1:1 ratio. Next, the PCR amplicons were purified using VAHTS^TM^ DNA Clean Beads (N411-03, Vazyme, China) and magnetic beads. The amplicons were then subjected to a second electrophoresis and purified from agarose gels using the Monarch^®^ DNA Gel Extraction Kit (250 steps, New England Biolabs, China). Quantification of the total PCR amplicons was performed using the Qubit^®^ dsDNA HS Assay Kit and a Qubit^®^ 4.0 Fluorometer (Invitrogen, Thermo Fisher Scientific, USA). Finally, libraries were prepared from the amplified DNA using the SMRTbell^TM^ Express Template Prep Kit (v2.0, Pacific Biosciences, USA) according to the manufacturer's instructions.

### High throughput sequencing

Single-molecule real-time (SMRT) sequencing was performed on the PCR amplification products of 30 samples using the Sequel II sequencing system (Pacific Biosciences, USA). After sequencing, effective circular consensus sequences (CCS) were obtained by performing quality control on the sequence data. The raw sequence data have been submitted to the NCBI Sequence Read Archive (SRA) under the accession numbers PRJNA1080264 and PRJNA1081168.

### Data processing

The effective CCS sequences were clustered using Usearch v10.0.240_i86 at a 97% similarity level, yielding OTUs and representative sequences after normalizing the sample sequences to the minimum number. Based on the OTUs classification data, a histogram of OTUs distribution across different samples was generated using Matplotlib v1.4.3. Using QIIME 2 v2020.6 (Evan et al., 2019), the “qiime diversity alpha-rarefaction” command and corresponding scripts were employed to calculate the alpha diversity index for each sample. The significance of index differences between groups was assessed using Student's t-test. A boxplot of the results was then generated using R (Picante v1.8.2).

The representative OTUs sequences were annotated using the RDP Classifier v2.7 based on the Unite 8.0 ITS fungal taxonomy database to obtain species classification information at different taxonomic levels. Species abundance tables were generated at various taxonomic levels using QIIME 2 v2020.6.0, and differences in species abundance among groups were tested using the F-test. Subsequently, R (ggplot v3.1.1) was used to draw bar charts illustrating species distribution and significant differences at the phylum and genus levels.

Principal coordinates analysis (PCoA) was conducted on the classification data using R (vegan v2.3-0), with species differences and abundance at the phylum and genus levels weighted according to the Bray-Curtis algorithm. Significance and correlation matrices between groups were calculated using Pearson's correlation and the pairwise test of permutational multivariate analysis of variance (PERMANOVA). PCoA and intergroup distance matrix plots were generated at the phylum and genus levels using R (ggplot).

The FUNGuild v1.0 tool was employed to classify and test the differences in functional genes of fungal communities, followed by clustering species according to their ecological functions.

The Varpart function in R (vegan v2.3-0) was used to analyze the explanatory ratios of three types of biological factors, including Nutrient, Aminosugar, and Plantfiber, on changes in community structure. The correlation between biological factors and species was analyzed using Redundancy Analysis (RDA) or Canonical Correspondence Analysis (CCA), with significant relationships marked by Pearson correlation coefficients.

### Detection of biological factors

Total Carbon (TC) in leaf was determined using the potassium dichromate volumetric method. Total Nitrogen (TN) was measured using the Micro-Kjeldahl method, and Total Phosphorus (TP) was determined by molybdenum antimony colorimetry. A flame photometer was used to measure Total Potassium (TK). Cellulose, hemicellulose, and lignin were quantified via ELISA using a Content Assay Kit (Michy Biomedical Technology, China). Glucosamine (GluN) and Galactosamine (GalN) were detected by liquid chromatography using HPLC (Agilent 1100, Agilent Technologies Inc., USA).

## Results and analysis

### Diversity differences of fungal communities in different decomposition periods of leaf litter based on OTUs

**Figure 1:**
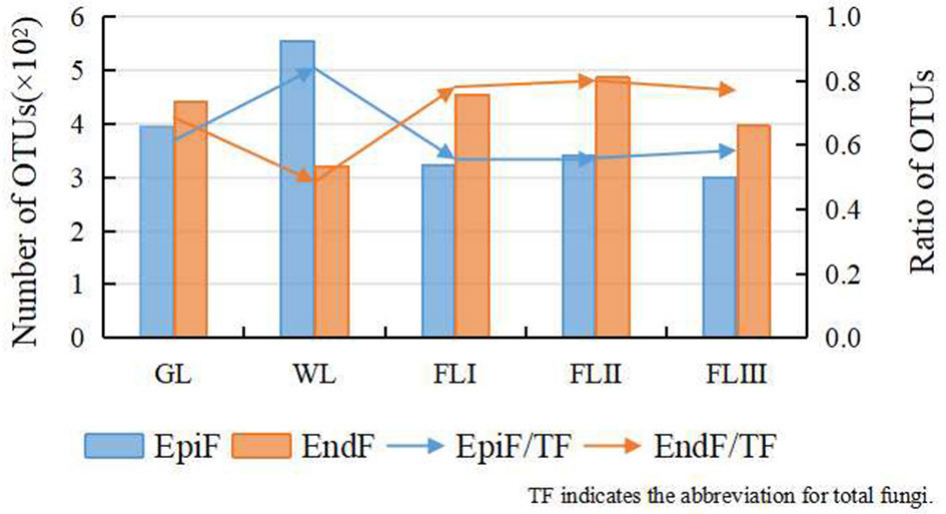
OTUs cluster distribution of the fungi during different periods of leaf litter decomposition.

After quality control, 1,774,385 effective CCS sequences were obtained, resulting in the clustering of 1,263 OTUs. As shown in Figure 1, following leaf litter decomposition, EndF OTUs briefly decreased during the WL phase but gradually increased throughout the decomposition process, while EpiF OTUs steadily declined. The WL phase acted as a transition point for the shift in both fungal living environments, with EndF OTUs emerging as the primary contributors to decomposition.

**Figure 2:**
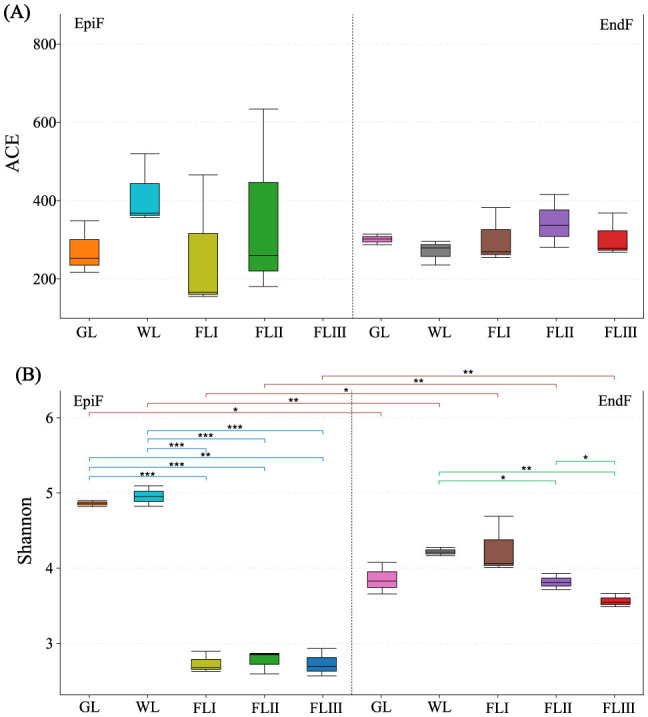
Alpha diversity indices of fungal communities based on OTUs during different decomposition periods of larch leaf litter, ACE **(A)** and Shannon **(B)**.

In this study, when the fungal library coverage exceeded 0.9999 in each sample, the results of the Alpha diversity analysis were deemed representative of the true fungal communities on leaf litter. As illustrated in Figure 2A, the ACE index of EpiF was highest in WL, while for EndF, it was highest in FLII. This indicates that phyllospheric fungi not only had high abundance but also a relative advantage for rare species in WL and FLII. Figure 2B shows that the Shannon index of EpiF in GL and WL was higher than in FL, indicating that the fungal community of larch needles had high Alpha diversity before leaf fall. However, Alpha diversity decreased significantly (0.000041 < *P* < 0.0017) once the leaf fell into the soil (FLI, FLII, FLIII). The Shannon index of EndF gradually decreased in the following order: WL > FLI > FLII > FLIII, suggesting that the diversity of EndF declined as the decomposition process progressed (0.0012 < *P* < 0.037). In contrast, the Shannon index of EpiF was higher than that of EndF in GL and WL. After reaching the FL period, although the diversity of EndF continued to decline, it remained higher than that of EpiF. Additionally, the Index Diversity Test revealed significant or highly significant differences (0.0011 < *P* < 0.012) in species abundance and evenness (as represented by the Shannon index) between EpiF and EndF within the same decomposition period. Particularly, although the evenness of both fungi showed a significant downward trend during soil decomposition, there were still significant differences in Alpha diversity. In summary, endophytic fungi displayed higher overall diversity at all decomposition stages, indicating a potentially stronger role in long-term litter nutrient cycling.

### Composition and distribution of major fungal communities in different decomposition periods of leaf litter

**Figure 3:**
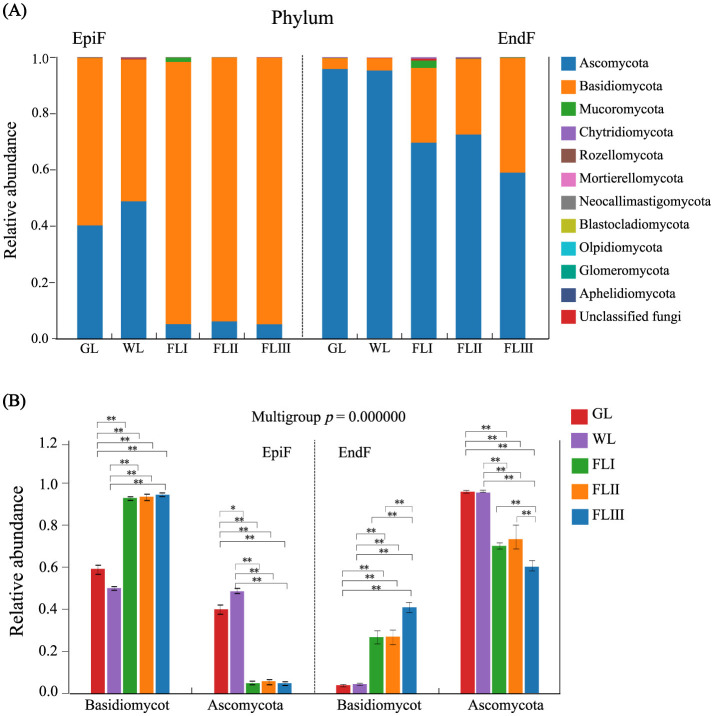
**(A)** Composition of fungal communities and **(B)** significance test of intergroup differences for dominant fungi at the phylum level during different decomposition periods of larch leaf litter.

As shown in Figure 3A, 11 known fungal phyla, including Ascomycota, Basidiomycota, Mucoromycota, Chytridiomycota, and Rozellomycota, were detected during the decomposition of larch leaf litter in the Greater Khingan Range. Basidiomycota was the dominant phylum in the epiphytic environment. As shown in Figure 3B, The relative abundance of Basidiomycota significantly increased from 0.595 in GL and 0.504 in WL to between 0.932 and 0.948 in FLI, FLII, and FLIII, with an extremely significant difference (*P* < 0.001). Ascomycota, a relatively dominant phylum, had a higher abundance in GL (0.402) and WL (0.488), but showed a significant downward trend (*P* < 0.001) in FL, dropping to between 0.051 and 0.061. In the endophytic environment, Ascomycota was the dominant phylum. Its abundance was higher in GL (0.958) and WL (0.954), but then decreased significantly (*P* < 0.001) during FL decomposition, ranging from 0.590 to 0.725. Basidiomycota, a relatively dominant phylum, showed an increase in abundance (0.0387 to 0.407) as the decomposition process progressed. Overall, the abundance of Basidiomycota and Ascomycota fluctuated dynamically, exhibiting a “rise-and-fall” relationship as the decomposition process progressed. The dominance of Basidiomycota among epiphytic fungi in the GL and WL phases suggests their role as initial colonizers during the early stages of litter decomposition, while the prevalence of Ascomycota in endophytic fungi indicates their primary role in the later stages of decomposition.

**Figure 4:**
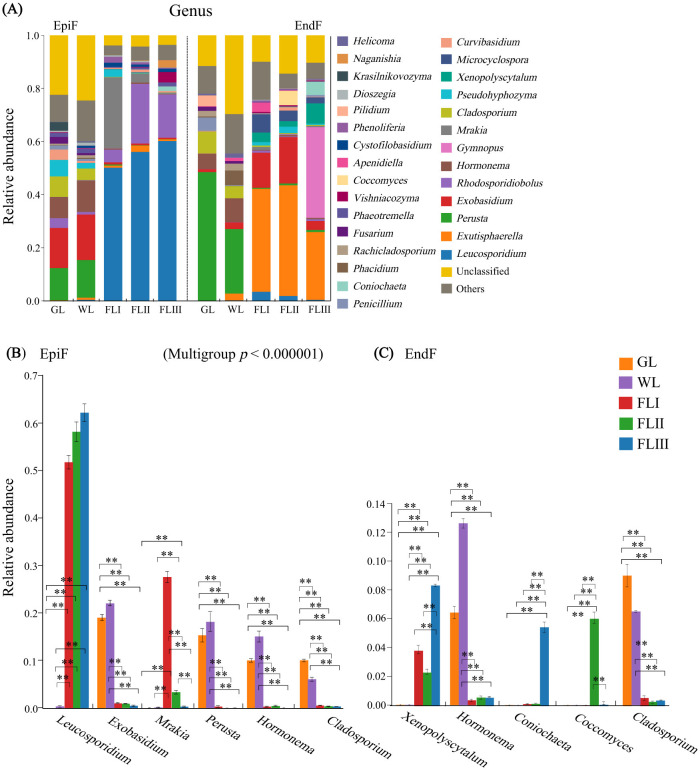
**(A)** Composition of fungal communities, **(B, C)** significance test of intergroup differences for dominant fungi at the genus level during different decomposition periods of larch leaf litter.

The bar chart in Figure 4A shows the fungal genera with a relative abundance greater than 0.003. In the epiphytic environment, 11 genera, including *Leucosporidium, Rhodosporidiobolus, Exobasidium, Mrakia, Perusta, Hormonema*, and *Cladosporium*, were dominant with a relative abundance exceeding 0.01. *Leucosporidium* (0.333) was the absolute dominant genus. The dominant genera shown in Figure 4B displayed significant differences in abundance across different decomposition periods of leaf litter. Notably, the abundance of *Leucosporidium* significantly increased from GL to WL and then to FL (*P* < 0.01), while the abundance of *Exobasidium, Perusta, Hormonema*, and *Cladosporium* significantly decreased after the leaf fell to the soil (*P* < 0.001). In the endophytic environment, 15 genera, including *Exutisphaerella, Perusta, Exobasidium, Gymnopus, Hormonema, Cladosporium*, and *Xenopolyscytalum*, were dominant with relative abundance > 0.01. Among them, *Exutisphaerella* (0.211) and *Perusta* (0.146) were the absolute dominant genera, with their abundance gradually increasing and decreasing, respectively, as the leaf decomposed. As shown in Figure 4C of the intergroup difference test results, the abundance of *Hormonema* and *Cladosporium* significantly decreased from 0.073 and 0.063 to 0.004 and 0.004, respectively, after leaf fall (*P* < 0.001), while *Xenosporum* significantly increased from 0.0001 to 0.043 (*P* < 0.001). In summary, the composition and abundance of dominant fungal genera in leaf litter varied not only across different decomposition periods but also showed significant differences in the enrichment of dominant fungi between epiphytic and endophytic environments. While *Leucosporidium* continued to proliferate in the epiphytic environment during the later stages of decomposition, the defensive capabilities of *Exutisphaerella* against *Leucosporidium* and *Perusta* attacks in the endophytic environment provided it with a sustained advantage for invasion.

### Beta diversity of fungal communities during different decomposition periods of leaf litter

**Figure 5:**
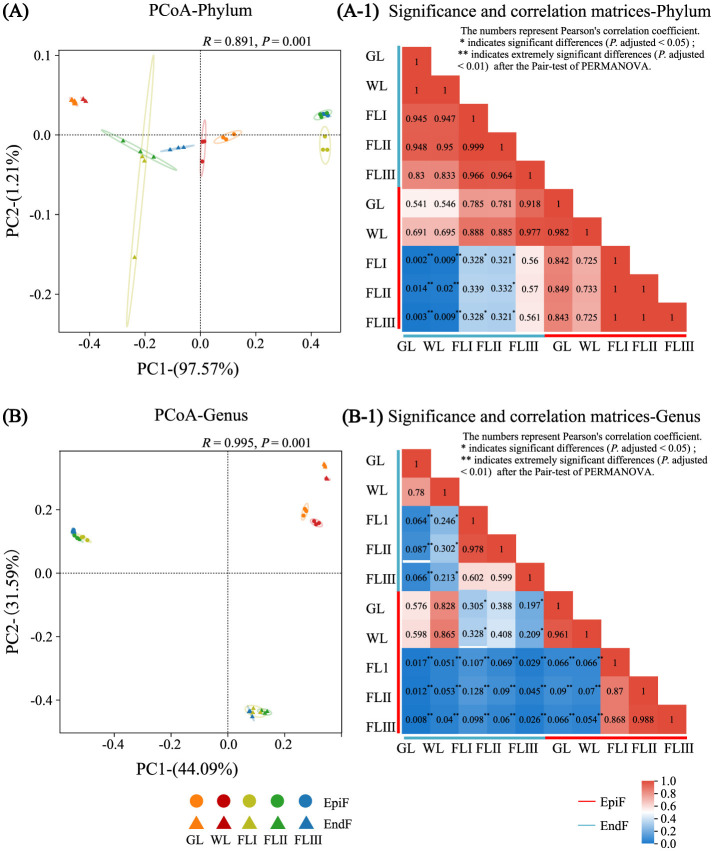
PCoA analysis, significance, and correlation matrix of the fungal communities during different decomposition periods of larch leaf litter at the genus **(A, A-1)**, phylum **(B, B-1)**.

As shown in Figure 5, based on the premise that the contribution value of the fungal community difference in the first principal component at the phylum level was 97.57%, the PCoA (Figure 5A) and the heatmap (Figure 5A-1) revealed a high similarity of phyllospheric fungi across GL, WL, FLI, FLII, and FLIII, with similarity coefficients ranging from 0.725 to 1 for EpiF and from 0.945 to 1 for EndF. Notably, the similarity coefficients among EpiF in FLI, FLII, and FLIII, and EndF between GL and WL, reached 1, indicating a high similarity of fungal communities at the phylum level during different decomposition periods of leaf litter.

However, the diversity of communities at the genus level was more complex. As shown in the PCoA chart in Figure 5B and the heatmap in Figure 5B-1, the community similarity coefficients were 0.961 for EpiF and 0.780 for EndF between GL and WL. Among FLI, FLII, and FLIII, the coefficients ranged from 0.868 to 0.988 for EpiF and from 0.599 to 0.978 for EndF. The distances between confidence ellipses were relatively close, with significant overlap, indicating a high similarity of fungal communities between GL and WL, as well as among FLI, FLII, and FLIII. However, the similarity coefficients of fungi before leaf fall (GL, WL) ranged from 0.054 to 0.090 for EpiF and from 0.064 to 0.302 for EndF after leaf fall (FLI, FLII, FLIII), with confidence ellipses being far apart, indicating significant differences in intergroup pair tests. These results suggest that the leaves falling into the soil acted as a “watershed,” leading to differences in fungal genera during various decomposition periods. In the later stages of decomposition, epiphytic fungi faced external invasions, while endophytic fungi adapted their life strategies to the micro-environment within the leaves. This dynamic resulted in the establishment of new diversity systems for both epiphytic and endophytic fungi, creating a “community watershed” between the early and late stages of decomposition. Additionally, compared to GL and WL, the similarity coefficients between EpiF and EndF were 0.107 in FLI, 0.090 in FLII, and 0.026 in FLIII, reflecting significant community differences. This indicates that the survival site of fungi in leaf litter was a crucial environmental factor influencing community differences during decomposition, directly leading to significant intergroup differences (*P* adjusted <0.05). This discrepancy was often related to the life histories of epiphytic and endophytic fungi in the later stages of decomposition. After the needles withered, native fungi gradually lost their ability to shape the foliage, facing an urgent need for survival, which resulted in heterogeneous colonization patterns of fungi in both epiphytic and endophytic environments.

### Main ecological types of fungal communities during the decomposition of leaf litter

**Figure 6:**
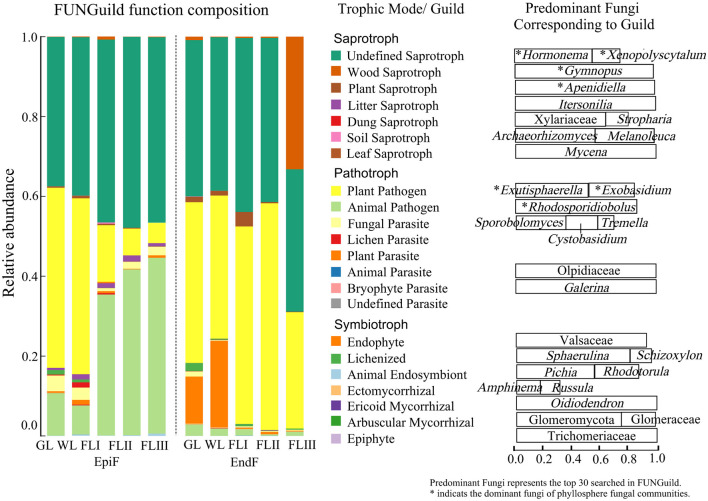
Ecological functions of fungal communities during the decomposition of leaf litter. The predominant fungi were defined as the top 30 genera identified using FUNGuild. An asterisk (*) indicates the dominant fungi within the phyllosphere fungal communities.

**Figure 7:**
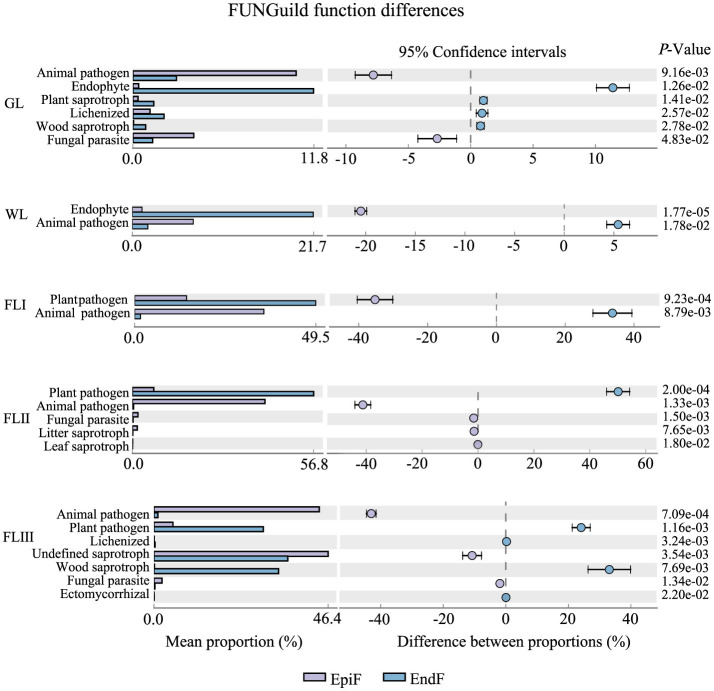
Ecological functional differences of the fungal communities during different decomposition periods of leaf litter.

As shown in Figure 6, during the decomposition of larch leaf litter in the Greater Khingan Range, the fungal communities were categorized into three main nutritional modes: Symbiotroph, Pathotroph, and Saprotroph. The environmental resource utilization patterns (guild) differed among these fungal groups. The predominant guilds (with relative abundance > 0.1) for EpiF were Undefined Saprotroph, Animal Pathogen, and Plant Pathogen, whereas the main guilds for EndF were Plant Pathogen and Undefined Saprotroph. From the differences in the guilds of the different fungi (Figure 7), Animal Pathogens, Fungal Parasites, and Plant Saprotrophs were widespread during the leaf litter decomposition periods, with significant differences between the guilds of EpiF and EndF. Specifically, Animal Pathogens and Fungal Parasites were significantly more abundant in the guild of EpiF, whereas Plant Saprotrophs were significantly more abundant in EndF (*P* < 0.05). Moreover, there were distinct distribution differences in the utilization of fungal resources during the different decomposition periods. For instance, Endophytes were significantly abundant in both GL and WL of EndF, Litter Saprotrophs and Leaf Saprotrophs were significantly abundant in FLII of EpiF, and Wood Saprotrophs and Ectomycorrhizal fungi were significantly abundant in FLIII of EndF.

By clarifying the guilds and distribution differences of phyllospheric fungi, we also found that the dominant fungal communities can utilize similar environmental resources through comparable survival strategies (Figure 6). For example, fungi such as *Hormonema, Xenopolyscytalum, Gymnopus*, and *Apenidiella* obtain nutrients by degrading or damaging dead host cells (Saprotrophs), whereas *Exutisphaerella, Exobasidium*, and *Rhodosporidiobolus* exploit material resources by causing host diseases (Pathotrophs). Therefore, Saprotrophs and Pathotrophs were the primary ecological types through which dominant fungi contributed to the decomposition of leaf litter.

### Changes in biological factors and their correlation with fungal communities in the decomposition of leaf litter

As shown in Table 1, the decomposition of leaf litter led to significant changes in various biological factors. TN, TP, and TK levels decreased significantly as decomposition progressed, while TC gradually increased, with a particularly notable enrichment of carbon after leaf fall. Regarding the leaf fiber structure, the cellulose quality remained stable and was slightly lower in FLIII, but did not show a significant decrease across the decomposition periods. Hemicellulose, on the other hand, remained stable during the WL and FLI, but significantly increased in content during FLII and FLIII as cellulose depolymerized. Lignin, which acts as a robust matrix for the leaf structure, did not exhibit significant loss until the FLIII. In terms of metabolic byproducts, GluN and GalN play key roles in microbe-mediated leaf decomposition. The residual concentration of GluN in the leaf increased significantly with further decomposition, whereas GalN levels did not follow a consistent pattern but did show a significant increase in FLIII. It can be seen that although the experiment involved the one-year decomposition of leaf litter, the changes in biological factors still reflected a significant dynamic decomposition process of the leaf matrix.

**Table 1:** Biological factors of larch leaf litter during different decomposition periods.

**Sample**	**Biological factors (mean** ±**SE)**									
	**TC (mg/g)**	**TN (mg/g)**	**TP (mg/g)**	**TK (mg/g)**	**C/N**	**Cellulose (mg/g)**	**Hemicellulose (mg/g)**	**Lignin (mg/g)**	**GluN (**μ**g/g)**	**GalN (**μ**g/g)**
**GL**	429.23 ± 2.26c	16.74 ± 0.16a	3.01 ± 0.04a	3.78 ± 0.04a	25.65 ± 0.11d	391.15 ± 8.91a	263.33 ± 7.48ab	165.25 ± 1.72a	876.15 ± 4.48c	19.00 ± 0.04bc
**WL**	432.68 ± 1.23bc	5.70 ± 0.13d	2.76 ± 0.07b	3.28 ± 0.03b	76.07 ± 1.94a	382.90 ± 5.3a	262.05 ± 1.85b	165.99 ± 2.42a	724.51 ± 5.66e	13.44 ± 0.53d
**FLI**	456.44 ± 2.03a	7.62 ± 0.19c	1.74 ± 0.01c	2.64 ± 0.10c	59.97 ± 1.39b	389.34 ± 6.45a	258.84 ± 4.55b	164.36 ± 3.25a	822.84 ± 6.78d	19.25 ± 0.25b
**FLII**	454.32 ± 1.52a	8.28 ± 0.03b	1.60 ± 0.02d	2.62 ± 0.01cd	54.90 ± 0.36c	386.73 ± 6.22a	280.73 ± 4.95a	169.02 ± 4.91a	930.95 ± 7.62b	17.48 ± 1.10bc
**FLIII**	444.60 ± 1.31b	8.19 ± 0.08b	0.59 ± 0.01e	2.44 ± 0.05d	54.31 ± 0.40c	380.33 ± 9.84a	274.05 ± 8.05ab	139.74 ± 6.46b	1,158.77 ± 4.21a	55.05 ± 0.36a

TC, Total Carbon; TN, Total Nitrogen; TP, Total Phosphorus; TK, Total Potassium; C/N, The ratio of C to N; GluN, Glucosamine; GalN, Galactosamine.

**Figure 8:**
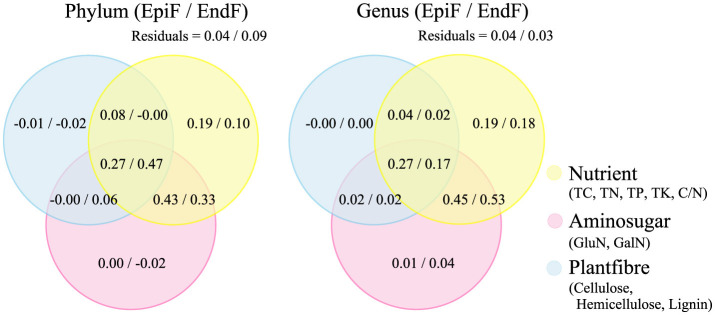
VPA of the contribution of biological factors to fungal community structure.

The Venn diagrams in Figure 8 illustrate the outcomes of a variance partitioning analysis (VPA) that categorizes biological factors (TC, TN, TP, TK, C/N, GluN, GalN, cellulose, hemicellulose, and lignin) into three groups: Nutrient, Aminosugar, and Plantfiber. The VPA illustrates the contributions of these biological factors, as well as spatial adjacency factors and their interactions, to fungal community structure. At both the phylum and genus levels, the explanatory ratio of Nutrient alone accounted for 0.10–0.19 of the variance in fungal community changes. The combined effect of Nutrient and Aminosugar explained 0.33–0.53 of the variance, while the interaction between Nutrient, Aminosugar, and Plantfiber accounted for 0.17–0.47. This indicates that the combined influence of these biological factors has a substantial explanatory power for changes in fungal community structure. The proportion of unexplained variance (residuals) was relatively low, ranging from 0.03 to 0.09. The VPA confirms that the biological factors considered in this study significantly impact the decomposition process of phyllospheric fungi in leaf litter.

After clarifying the close relationship between biological factors and fungal involvement in leaf litter decomposition, RDA/CCA and significance correlation analyses further revealed the contribution and relevancy of major fungi to decomposition at the phylum and genus levels.

**Figure 9:**
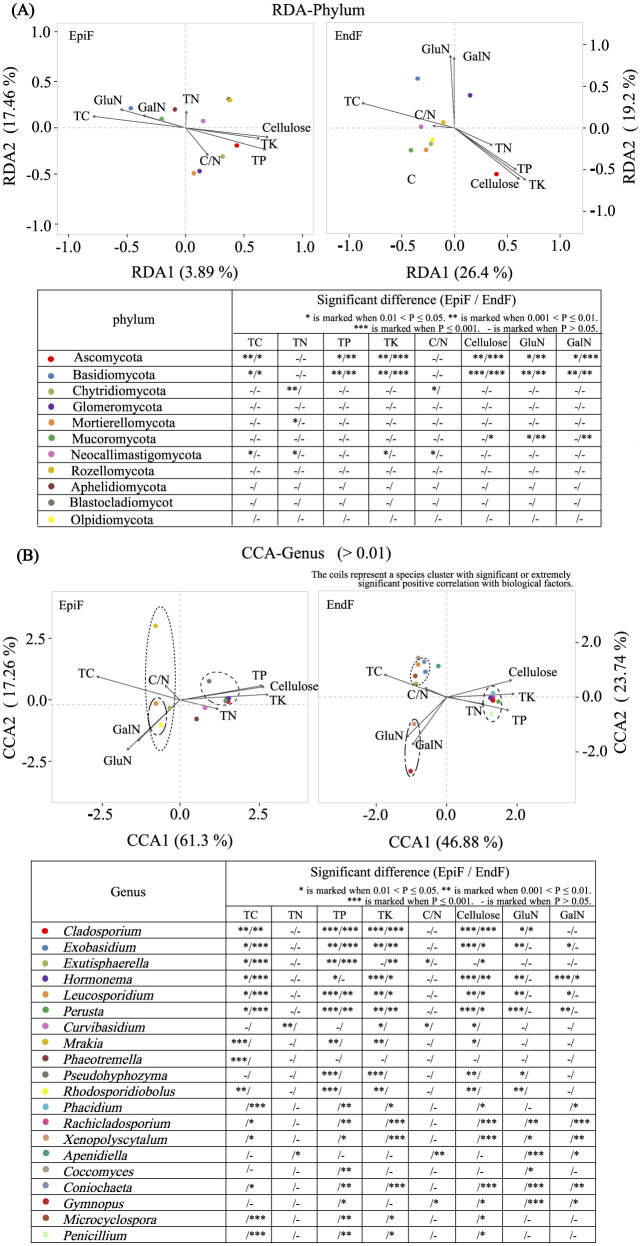
**(A)** RDA for Phylum/**(B)** CCA for genus analysis of the relationship between fungi and biological factors.

Figure 9A shows that despite environmental differences between EpiF and EndF, the dominant phyla (Basidiomycota and Ascomycota) exhibited consistent significant correlations with biological factors in both environments. For example, Basidiomycota exhibited significant or extremely significant positive correlations with TC, GalN, and GluN, and extremely significant negative correlations with TP, TK, and cellulose in epiphytic and endophytic environments. Conversely, Ascomycota showed opposite correlations with these biological factors compared to Basidiomycota. The observed trends in Aminosugar indicators suggest that Basidiomycota is a major contributor to organic matter transformation and accumulation in leaf litter decomposition, whereas Ascomycota plays a crucial role in cellulose breakdown and phosphorus and potassium enrichment.

Figure 9B presents the correlation analysis between dominant genera (relative abundance > 0.01) and biological factors. For EpiF, *Leucosporidium* and *Rhodosporidiobolus* were the absolute dominant genera, showing significant or extremely significant positive correlations with TC, GalN, and GluN. Other low-abundance genera in EpiF, such as *Mrakia* and *Exutisphaerella*, exhibited significant positive correlations with TC, whereas *Exobasidium, Perusta, Hormonema, Cladosporium*, and *Pseudohyphzyma* were positively correlated with TP, TK, and cellulose. *Curvibasidium* showed an extremely significant positive correlation with TN. For EndF, significant or extremely significant positive correlations were observed between *Exutisphaerella, Exobasidium, Microcyclospora, Leucosporidium, Coniochaeta*, and TC; between *Gymnopus* and *Xenopolyscytalum* with GalN and GluN; and between *Perusta, Hormonema, Cladosporium, Phacidium, Rachicladosporium*, and *Penicillium* with TP, TK, and cellulose. The significant clustering of fungal genera with biological factors was evident (Figure 9B), underscoring the role of these genera in leaf litter decomposition. The leaching of TC, TN, TP, and TK, the decomposition of supramolecular compounds such as cellulose, and the metabolism of biological polysaccharides such as GalN and GluN were key biological factors influencing the distribution of dominant fungal genera involved in leaf litter decomposition.

## Discussion

### Leaf litter decomposition affects the diversity of the phyllospheric fungal community

Previous studies have hypothesized that there is a considerable diversity overlap between living leaf fungi and leaf litter fungi based on phylogenetics, cluster analysis, and non-metric multidimensional scaling (Unterseher et al., 2013). Our study, however, found no significant difference in alpha diversity between phyllospheric fungi of GL and WL. Beta diversity analysis also revealed a high similarity in fungal community structure between GL and WL. In contrast, there were significant differences between the fungal communities of GL, WL, and FL. Thus, the previous hypothesis may only apply to the leaf withering period prior to leaf fall. Healthy leaves provide a relatively stable microenvironment for microorganisms, where most phyllospheric fungi rely on readily available sugars and other compounds for survival. After leaf death, while these compounds are rapidly consumed, the original phyllospheric fungi can temporarily maintain species diversity using residual nutrients until the leaf has fully decomposed. The significant difference in fungal diversity between withered and fallen leaves is partly due to the invasion of soil fungi into the fallen leaf, which predominantly affects EpiF and has a delayed effect on EndF. Another key factor is that the phyllotropic fungi lost “biological buffering system” of plant tissue after leaf fall (Hofstetter et al., 2012). As the change of leaf tissue environment, the litter fungi had to establish a new diversity system by “survival of the fittest”, leading to substantial changes in the fungal communities from withered to fallen leaf.

In addition to the differences in fungal diversity across decomposition periods, there were notable differences between EpiF and EndF fungal communities. The number of OTUs for EndF was slightly higher than that for EpiF on healthy leaf, although the diversity index for EpiF was higher. After leaf withering, EndF experienced a sharp decline in OTUs due to the rapid depletion of leaf nutrients but showed recovery upon infection by saprophytic fungi. Following leaf fall, the competitive advantage of EndF in decomposition resulted in a significant decline in OTUs and diversity of EpiF, while the lag of host resource utilization by EpiF resulted in a higher species diversity of EndF. EndF maintained higher species diversity after leaf fall due to its environmental advantage of preferentially settling in leaf and utilizing potential saprophytic capabilities more effectively (U'Ren and Arnold, 2016). Additionally, EndF may have an adaptive ability to produce defensive chemicals in leaf litter (Jia et al., 2020). In contrast, EpiF had limited growth capacity in living host tissues and often died after leaf aging due to functional trade-offs (Jeewon et al., 2018). Thus, EndF constitutes a crucial part of the litter fungi community and is the most active fungal group in annual leaf litter decomposition.

### Heterogenous colonization of EpiF and EndF in the phyllospheric microenvironment

Previous theories on fungal succession in litter decomposition have suggested that not all fungi inhabiting withered leaf transition to fallen leaves and that endophytic fungi in healthy leaves might act as pioneers in decomposing decaying plants (Osono and Hirose, 2009). According to this theory, Ascomycota, the dominant phylum of EndF in both green and withered leaf in this study, would readily occupy this pioneer decomposition niche. However, as decomposition progressed, the endophytic environment dominated by Ascomycota was invaded by Basidiomycota. Some researchers believe that Ascomycota and other phyla are generally less capable of decaying litter than Basidiomycota, especially as the demand for leaf litter decay increases (Fukasawa et al., 2009). This view is supported by our study, which observed trends in the abundance of dominant Basidiomycota genera during leaf decomposition. For instance, genera such as *Leucosporidium, Rhodosporidiobolus, Phenoliferia*, and *Naganishia*, which originated from healthy leaves, continued to proliferate in the epiphytic environment of FL. Notably, *Exobasidium* shifted from being epiphytically predominant in GL to endophytically predominant in FL. Conversely, non-Basidiomycota genera such as *Perusta, Hormonema*, and *Cladosporium* gradually lost their abundance as decomposition intensified. Despite the expansion of Basidiomycota within the phyllosphere, Ascomycota genera such as *Exutisphaerella, Xenopolyscytalum, Coccomyces*, and *Coniochaeta* still maintained population dominance, indicating that Ascomycota can strongly defend against saprophytic Basidiomycota (Fukasawa et al., 2009). However, this population dominance was limited to the endophytic environment.

We believe that the heterogeneous colonization of phyllospheric fungi mentioned above is related to their life history. In the natural environment, phyllospheric fungi persist on healthy leaf for long periods. As leaves wither and age rapidly due to changes in host age and climate, phyllospheric fungi trigger saprophytic or pathogenic metabolic processes in a short time, accelerating leaf death (Unterseher et al., 2013). After the leaf fall, these saprophytic or pathogenic fungi reproduce spores that reinfect the endophytic leaf tissues, thereby entering the litter decomposition cycle (Sun et al., 2011). The evolutionary history of endophytic fungi also supports the commonly held view that endophytic fungi evolved from saprophytic or pathogenic fungi through multiple parallel and opposite trajectories since the early evolution of terrestrial plants (Saikkonen et al., 2015). Our study on the ecological roles of phyllospheric fungi revealed that dominant fungi such as *Exutisphaerella, Exobasidium*, and *Rhodosporidiobolus* proliferated by degrading dead host cells or causing host diseases to obtain nutrients during leaf decomposition. Increasing evidence suggests that as leaves age and die, native fungi lose their ability to shape the foliage and adapt to these unbuffered microhabitats by altering their life strategies. These life-strategy-altering saprophytic or pathogenic fungi are likely the first colonizers involved in the early periods of leaf litter decomposition (Weatherhead et al., 2022).

### Leaf litter nutrient loss and its reciprocal feedback with fungal population

In our study on larch leaf litter in the Cold Temperate Zone, we found that the degree of cellulose decomposition was positively correlated with the loss of N, P, and K as leaf litter decomposition intensified. Hemicellulose, as an intermediate product of cellulose decomposition, exhibited varying degrees of negative correlation with N, P, and K as cellulose deconstruction progressed. These results further confirmed a strong correlation between leaf fiber loss and nutrient return, which is influenced not only by the nutrient enrichment and release modes of leaf litter (Wu et al., 2024; Chen et al., 2023) but also by the reciprocal feedback between leaf nutrients and fungal populations.

Our study found that the combined effects of biological factors such as C, N, P, K, and cellulose had a high explanatory power for the changes in fungal community structure during leaf litter decomposition. These factors were the primary drivers of species differences, as verified by the clustering effects observed between key fungal genera groups and biological factors.

Some studies have suggested that the continuous secretion of GluN serves as a marker of intensified leaf litter decomposition (Yang et al., 2019). The CCA showed that genera such as *Gymnopus, Xenopolyscytalum, Leucosporidium*, and *Rhodosporidiobolus* not only continued to proliferate in the later stages of decomposition but also exhibited a strong positive correlation with GluN synthesis. This indicates that the activity of these fungi was enhanced during the late stages of leaf decay. GluN is widely regarded as an “activator” for labeling microbial residual carbon, suggesting that these fungi play an important role in mediating the carbon cycle of leaf litter during decomposition. From the perspective of the association between key fungal communities and nutrients, *Basidiomycota* and genera such as *Leucosporidium, Rhodosporidiobolus, Exutisphaerella*, and *Exobasidium* demonstrated a stronger association with carbon. These were key fungal clusters responsible for carbon fluctuations and significant contributors to the transformation and accumulation of organic matter during leaf litter decomposition. These fungal communities effectively decomposed lower recalcitrant components, such as oligosaccharides, organic acids, and phenolic compounds in the early stages of decomposition, making them key participants in the mineralization of biomass carbon in northern forests, as confirmed by studies on the litter decomposition function of *Basidiomycota* (Baldrian, 2017; Purahong et al., 2016).

Moreover, *Ascomycota*, along with genera such as *Exobasidium, Perusta, Hormonema*, and *Cladosporium*, played significant roles in cellulose deconstruction and the enrichment of P and K. Previous studies have shown that *Ascomycota* have a dominant advantage in the early decomposition of forest litter due to their colonization order (Hoppe et al., 2016). They can effectively break down substantial amounts of cellulose and hemicellulose in litter cell walls (Riley et al., 2014). We believe that the cellulose degradation function of *Ascomycota* and related fungal communities is triggered by late-stage saprophytic metabolic processes. These fungi “attack” cellulose and hemicellulose microfibril structures by releasing endo-glucanases, breaking down specific macromolecules into smaller, soluble molecules. Thus, in order to degrade the litter substrate gradually, the fungal community structure must undergo population succession during the decomposition process to meet metabolic requirements.

In addition, many scholars believed that fungal communities such as *Fusarium, Penicillium*, and *Alternaria* exhibit high activity in producing leaf decomposition enzymes (Zhang et al., 2023) and are key contributors to cellulose decomposition (Zheng et al., 2021). Other studies have also confirmed the existence of a broad core functional cluster in the phyllosphere (Sun et al., 2021), which has notable synergistic effects in promoting litter decomposition (Ruiz-Prez et al., 2016). Although our study provided evidence supporting the theory of functional core fungal clusters in leaf litter, our understanding of these phenomena at the regional level remains limited.

Further studies will be needed to gain insights into the molecular mechanisms underlying fungal decomposition of forest litter. Future studies should focus on the nutrient cycling pathways mediated by functional microbial communities. Additionally, a multidimensional discussion on the potential impacts of climate change on the dynamics of fungal communities in cold temperate ecosystems will be essential.

## Conclusions

In our study on the evolution of fungal communities and their relationship with nutrient loss in decomposing larch leaf litter in cold temperate forests, we observed significant heterogeneity in the colonization of epiphytic and endophytic fungi during decomposition. We discovered that endophytic fungi adapted their life strategies to gain a dominant advantage within the phyllospheric fungi community. Additionally, fungi were found to undergo population succession as the demand for litter decomposition intensified. Our findings confirmed that core functional fungal genera in the phyllosphere exhibited a synergistic effect on carbon fluctuations, cellulose deconstruction, and the enrichment of P and K in leaf litter. This study provides a more in-depth understanding of the role of phyllospheric fungi in litter decomposition and offers insights into the processes of boreal forest ecosystems and nutrient cycling. It also holds potential applications for assessing future global carbon dynamics and monitoring ecosystem stability in the context of climate change.

## Data Availability

The datasets presented in this study can be found in online repositories. The names of the repository/repositories and accession number(s) can be found in the article/supplementary material.

## References

[B1] Bahnmann B. Mašínová T. Halvorsen R. Davey M. L. Sedlák P. Tomšovsk,ý M.. (2018). Effects of oak, beech and spruce on the distribution and community structure of fungi in litter and soils across a temperate forest. Soil Biol. Biochem. 119, 162–173. 10.1016/j.soilbio.2018.01.021

[B2] Baldrian P. (2017). Forest microbiome: diversity, complexity and dynamics. FEMS Microbiol. Rev. 41, 109–130. 10.1093/femsre/fuw040 27856492

[B3] Carnicer J. Sardans J. Stefanescu C. Ubach A. Bartrons M. Asensio D.. (2015). Global biodiversity, stoichiometry and ecosystem function responses to human-induced C–N–P imbalances. J. Plant Physiol. 172, 82–91. 10.1016/j.jplph.2014.07.022 25270104 PMC6485510

[B4] Chakraborty K. Das A. R. Saha A. K. Das P. (2020). A culture based diversity of saprobic fungi associated with leaf litter of Hevea brasiliensis along a chronosequence of plantations in Tripura, Northeast India. Trop. Ecol. 61, 468–474. 10.1007/s42965-020-00104-7

[B5] Chen Y.-Q. Chen S.-F. Zhang B.-H. Ma X.-T. Liu X.-T. Huang Y.. (2023). Divergent decomposition patterns of leaf litter and fine roots from an urban forest in Mid-Subtropical China. Forests 14:1741. 10.3390/f14091741

[B6] Contos P. Murphy N. P. Kayll Z. J. Morgan T. Vido J. J. Decker O. (2024). Rewilding soil and litter invertebrates and fungi increases decomposition rates and alters detritivore communities. Ecol. Evol. 14:11128. 10.1002/ece3.11128 38469050 PMC10925487

[B7] Evan B. Ram R. J. Dillon M. R. Bokulich N. A. Abnet C. C. Al-Ghalith G. A.. (2019). Reproducible, interactive, scalable and extensible microbiome data science using QIIME 2. Nat. Biotechnol. 37, 852–857. 10.1038/s41587-019-0209-9 31341288 PMC7015180

[B8] Fukasawa Y. Matsukura K. Ando Y. Suzuki S. N. Okano K. Song Z.. (2021). Relative importance of climate, vegetation, and spatial factors in the community and functional composition of wood-inhabiting fungi in discontinuously distributed subalpine spruce forests. Can. J. Forest Res. 51, 1029–1038. 10.1139/cjfr-2020-0344

[B9] Fukasawa Y. Osono T. Takeda H. (2009). Effects of attack of saprobic fungi on twig litter decomposition by endophytic fungi. Ecol. Res. 24, 1067–1073. 10.1007/s11284-009-0582-9

[B10] Guo J. Yu L.-H. Fang X. Xiang W.-H. Deng X.-W. Lu X. (2015). Litter production and turnover in four types of subtropical forests in China. Acta Ecologica Sinica. 35, 4668–4677. 10.5846/stxb201312052896

[B11] Hofstetter V. Buyck B. Croll D. Viret O. Couloux A. Gindro K. (2012). What if esca disease of grapevine were not a fungal disease? Fungal Divers. 54, 51–67. 10.1007/s13225-012-0171-z

[B12] Hoppe B. Purahong W. Wubet T. Kahl T. Bauhus J. Arnstadt T.. (2016). Linking molecular deadwood-inhabiting fungal diversity and community dynamics to ecosystem functions and processes in Central European forests. Fungal Divers. 77, 367–379. 10.1007/s13225-015-0341-x

[B13] Jeewon R. Yeung Q. S. Y. Wannasinghe D. N. Rampadarath S. Puchooa D. Wang H.-K.. (2018). Hidden mycota of pine needles: molecular signatures from PCR-DGGE and Ribosomal DNA phylogenetic characterization of novel phylotypes. Sci. Rep. 8:18053. 10.1038/s41598-018-36573-z 30575771 PMC6303302

[B14] Jia Q. Qu J.-W. Mu H.-N. Sun H.-G. Wu C. (2020). Foliar endophytic fungi: diversity in species and functions in forest ecosystems. Symbiosis 80, 103–132. 10.1007/s13199-019-00663-x37973868

[B15] Leppert K. N. Niklaus P. A. Scherer-Lorenzen M. (2017). Does species richness of subtropical tree leaf litter affect decomposition, nutrient release, transfer and subsequent uptake by plants? Soil Biol. Biochem. 115, 44–53. 10.1016/j.soilbio.2017.08.007

[B16] Lin D.-M. Pang M. Fanin N. Wang H.-J. Qian S.-H. Zhao L.. (2019). Correction to: Fungi participate in driving home-field advantage of litter decomposition in a subtropical forest. Plant Soil. 444, 535–536. 10.1007/s11104-019-04210-x

[B17] Lin Z.-R. Chen L.-M. Dai Y. Chen S.-L. Li C.-J. Yuan T. (2024). Effects of enhanced UV-B radiation on decomposition and nutrient release rates of litter from Cunninghamia lanceolata (Lamb.) Hook. Forests 15:686. 10.3390/f15040686

[B18] Marian F. Brown L. Sandmann D. Maraun M. Scheu S. (2019). Roots, mycorrhizal fungi and altitude as determinants of litter decomposition and soil animal communities in tropical montane rainforests. Plant Soil. 438, 1–18. 10.1007/s11104-019-03999-x

[B19] Nakanishi R. Tsuyuzaki S. (2024). Litter decomposition rates in a post-mined peatland: determining factors studied in litterbag experiments. Environm. Proc. 11:2. 10.1007/s40710-024-00679-6

[B20] Osono T. Hirose D. (2009). Effects of prior decomposition of Camellia japonica leaf litter by an endophytic fungus on the subsequent decomposition by fungal colonizers. Mycoscience 50, 52–55. 10.1007/S10267-008-0442-4

[B21] Piazza M. V. Pinto P. Bazzoni B. Berenstecher P. Casas C. Zieher X. L. (2024). From plant litter to soil organic matter: a game to understand carbon dynamics. Front. Ecol. Environ. 22:2724. 10.1002/fee.2724

[B22] Purahong W. Wubet T. Lentendu G. Schloter M. Pecyna M. J. Kapturska D.. (2016). Life in leaf litter: novel insights into community dynamics of bacteria and fungi during litter decomposition. Molec. Ecol. 25:16: 10.1111/mec.13739 27357176

[B23] Riley R. Salamov A. A. Brown D. W. Nagy L. G. Floudas D. Held B. W.. (2014). Extensive sampling of basidiomycete genomes demonstrates inadequacy of the white rot/brown-rot paradigm for wood decay fungi. Proc. Natl. Acad. Sci. USA. 111, 9923–9928. 10.1073/pnas.1418116111 24958869 PMC4103376

[B24] Ruiz-Prez C. A. Restrepo S. Zambrano M. M. (2016). Microbial and functional diversity within the phyllosphere of Espeletia species in an Andean high- mountain ecosystem. Appl. Environ. Microbiol. 82, 1807–1817. 10.1128/AEM.02781-15 26746719 PMC4784022

[B25] Sagi N. Hawlena D. (2023). Climate dependence of the macrofaunal effect on litter decomposition-a global meta-regression analysis. Ecol. Lett. 27:14333. 10.1111/ele.14333 37874740

[B26] Saikkonen K. Mikola J. Helander M. (2015). Endophytic phyllosphere fungi and nutrient cycling in terrestrial ecosystems. Curr. Sci. 109, 121−126. Available at: https://www.jstor.orgstable/24905696

[B27] Song Y.-L. Xing J.-M. Hu C. Song C.-G. Wang Q. Wang S.-J. (2024). Decomposition and carbon and nitrogen releases of twig and leaf litter were inhibited by increased level of nitrogen deposition in a subtropical evergreen broad-leaved forest in Southwest China. Forests 15:492. 10.3390/f15030492

[B28] Sun X.-G. Zheng Y. Xu G. Guo Q.-Q. Tan J.-H. Ding G.-J. (2021). Fungal diversity within the phyllosphere of *Pinus massoniana* and the possible involvement of phyllospheric fungi in litter decomposition. Fungal Biol. 125, 785–795. 10.1016/j.funbio.2021.05.001 34537174

[B29] Sun Y. Wang Q. Lu X.-D. Okane I. Kakishima M. (2011). Endophytic fungal community in stems and leaf of plants from desert areas in China. Mycol. Prog. 11, 781–790. 10.1007/s11557-011-0790-x

[B30] Tian J. He N. P. Hale L. Niu S. L. Yu G. R. Liu Y.. (2018). Soil organic matter availability and climate drive latitudinal patterns in bacterial diversity from tropical to cold temperate forests. Funct. Ecol. 32, 61–70. 10.1111/1365-2435.12952

[B31] Unterseher M. Peršoh D. Schnittler M. (2013). Leaf-inhabiting endophytic fungi of European Beech (*Fagus sylvatica* L.) co-occur in leaf litter but are rare on decaying wood of the same host. Fungal Divers. 60, 43–54. 10.1007/s13225-013-0222-0

[B32] U'Ren J. M. Arnold A. E. (2016). Diversity, taxonomic composition, and functional aspects of fungal communities in living,senescent, and fallen leaf at five sites across North America. Peer J. 4:2768. 10.7717/peerj.2768 27994976 PMC5157190

[B33] Wang X. Ye R.-M. Li B.-L. Tian K. (2024). Emerging microplastics alter the influences of soil animals on the fungal community structure in determining the litter decomposition of a deciduous tree. Forests 15:488. 10.3390/f15030488

[B34] Waring B. G. (2013). Exploring relationships between enzyme activities and leaf litter decomposition in a wet tropical forest. Soil Biol. Biochem. 64, 89–95. 10.1016/j.soilbio.2013.04.010

[B35] Weatherhead E. Davis E. L. Koide R. T. (2022). Many foliar endophytic fungi of Quercus gambelii are capable of psychrotolerant saprotrophic growth. PLoS ONE. 17:0275845. 10.1371/journal.pone.0275845 36223398 PMC9555652

[B36] Wrightson I. Anaraki M. T. Uyl J. D. Nadelhoffer K. J. Lajtha K. Simpson M. J. (2024). Changes in litter and nitrogen deposition differentially alter forest soil organic matter biogeochemistry. Geochim. Cosmochim. Acta. 374, 186–199. 10.1016/j.gca.2024.04.030

[B37] Wu X.-J. Li J.-F. Jiang Y. Sun L.-J. Wu P.-F. Ma X.-Q. (2024). Effect of simulated warming on nutrient release during the decomposition of leaf litter and canopy litter of chinese fir based on displacement test. Acta Ecologica Sinica. 17, 1–12.

[B38] Wu Y. D. Mao W. L. Yuan Y. (2021). Comparison of polypore florae and diversity from temperate to subtropical forest zones in China. Biodivers. Sci. 29, 1369–1376. 10.17520/biods.202109434063014

[B39] Xing P.-F. Wang Y.-N. Lu X.-Y. Li H.-X. Guo J.-P. Li Y.-L.. (2024). Climate, litter quality and radiation duration jointly regulate the net effect of UV radiation on litter decomposition. Sci. Total Environ. 926:172122. 10.1016/j.scitotenv.2024.172122 38569973

[B40] Yan H.-Y. Gu X.-R. Shen H. (2010). Microbial decomposition of forest litter: a review. Chinese Journal of Ecology. 29, 1827–1835.

[B41] Yang L.-M. Chen S.-L. Li Y. Wang Q.-C. Zhong X.-J. Yang Z.-J.. (2019). Conversion of natural evergreen broadleaved forests decreases soil organic carbon but increases the relative contribution of microbial residue in subtropical China. Forests 10:468. 10.3390/f10060468

[B42] Zhang C.-F. De Pasquale Hartman K. Stanley C. E. Berendsen R. L. Van der Heijden M. G. A. (2023). The microbial contribution to litter decomposition and plant growth. Environ. Microbiol. Rep. 16:13205. 10.1111/1758-2229.13205 38018445 PMC10866077

[B43] Zhang J.-W. Du T. Liu S.-S. Abebe S. A. Yan S. Li W.. (2024). Carbon release characteristics at soil–air interface under litter cover with different decomposition degrees in the arbor and bamboo forests of Pi River Basin. Land 13:427. 10.3390/land13040427

[B44] Zheng H.-P. Yang T.-J. Bao Y.-Z. He P.-P. Yang K.-M. Mei X.-L.. (2021). Network analysis and subsequent culturing reveal keystone taxa involved in microbial litter decomposition dynamics. Soil Biol. Biochem. 157:108230. 10.1016/j.soilbio.2021.108230

